# Diverse antiviral IgG effector activities are predicted by unique biophysical antibody features

**DOI:** 10.1186/s12977-021-00579-9

**Published:** 2021-10-30

**Authors:** Hao D. Cheng, Karen G. Dowell, Chris Bailey-Kellogg, Brittany A. Goods, J. Christopher Love, Guido Ferrari, Galit Alter, Johannes Gach, Donald N. Forthal, George K. Lewis, Kelli Greene, Hongmei Gao, David C. Montefiori, Margaret E. Ackerman

**Affiliations:** 1 Thayer School of Engineering, Dartmouth College, Hanover, NH USA; 2 Molecular and Cellular Biology Program, Dartmouth College, 14 Engineering Dr., Hanover, NH 03755 USA; 3 Department of Computer Science, Dartmouth College, Hanover, 03755 USA; 4 Department of Chemical Engineering, Massachusetts Institute of Technology, Cambridge, MA 02139 USA; 5 Department of Biological Engineering, Koch Institute at MIT, Massachusetts Institute of Technology, Cambridge, MA 02139 USA; 6 Department of Surgery, Duke University Medical Center, Durham, NC 27710 USA; 7 Duke Human Vaccine Institute, Duke University Medical Center, Durham, NC 27719 USA; 8 Ragon Institute of MGH, MIT, and Harvard, Cambridge, MA 02139 USA; 9 Division of Infectious Diseases, Irvine School of Medicine, University California, Irvine, CA 92697 USA; 10 Division of Vaccine Research, Institute of Human Virology, University Maryland School of Medicine, Baltimore, MD 21201 USA

**Keywords:** IgG, Antibody, Effector function, HIV, Vaccine

## Abstract

**Background:**

The critical role of antibody Fc-mediated effector functions in immune defense has been widely reported in various viral infections. These effector functions confer cellular responses through engagement with innate immune cells. The precise mechanism(s) by which immunoglobulin G (IgG) Fc domain and cognate receptors may afford protection are poorly understood, however, in the context of HIV/SHIV infections. Many different in vitro assays have been developed and utilized to measure effector functions, but the extent to which these assays capture distinct antibody activities has not been fully elucidated.

**Results:**

In this study, six Fc-mediated effector function assays and two biophysical antibody profiling assays were performed on a common set of samples from HIV-1 infected and vaccinated subjects. Biophysical antibody profiles supported robust prediction of diverse IgG effector functions across distinct Fc-mediated effector function assays. While a number of assays showed correlated activities, supervised machine learning models indicated unique antibody features as primary contributing factors to the associated effector functions. Additional experiments established the mechanistic relevance of relationships discovered using this unbiased approach.

**Conclusions:**

In sum, this study provides better resolution on the diversity and complexity of effector function assays, offering a clearer perspective into this family of antibody mechanisms of action to inform future HIV-1 treatment and vaccination strategies.

**Supplementary Information:**

The online version contains supplementary material available at 10.1186/s12977-021-00579-9.

## Background

Passive administration of broadly neutralizing antibodies (bnAb) against HIV-1 has been shown to protect humanized mice [1], non-human primates (NHP) [2], and to reduce risk of infection by neutralization-sensitive viral strains in humans [3]. Robust protection conferred by broadly neutralizing antibodies has been widely recognized as the consequence of IgG variable domain (Fv) interactions with viral epitopes, via multiple mechanisms, including the block of viral attachment [4], inhibition of cell–cell transmission [5], and suppression of viremia [6]. The crystallizable fragment (Fc) part of the immunoglobulin (IgG) molecule, however, provides in vivo anti-viral effects as well, through engagement of innate immune Fcγ receptors (FcγRs). These activities contribute to bnAb-mediated anti-viral activity in some [7–10], but not all [11] studies that have looked to quantitatively evaluate their contribution, as recently reviewed [12].

Similarly, increasing evidence from animal models suggests that antibody effector functions also play a critical role in protective vaccine-mediated immunity. These activities include antibody-dependent phagocytosis [13], complement-dependent cytotoxicity (CDC) [14], antibody-dependent cellular viral inhibition (ADCVI) [15], and antibody-dependent cellular cytotoxicity (ADCC) [16], among others. Correlations between plasma ADCC activity and challenge resistance or decreased viral load have been observed in a number of NHP studies [17–20]. Antibody-dependent phagocytosis activity has also been reported to correlate with protection from infection in a growing number of preclinical studies [21, 22]. Polyfunctional antibody responses—those exhibiting the ability to elicit the anti-viral activities of diverse effector cell subsets, have also been reported to correlate with protection in vaccine studies [23]. The most definitive work to date that these correlations may represent mechanisms of protection comes from a passive transfer experiment in which polyfunctional but non-neutralizing antibodies were shown to protect NHP from SIV infection [24].

In humans, early work demonstrated the enrichment of ADCC-inducing antibodies in long-term HIV non-progressors [25–34]. Antibodies from HIV elite controllers have exhibited potentiated viral inhibition, NK activation, phagocytosis and antibody-dependent cellular cytotoxicity [35, 36]. Moreover, immune-escape variants have been identified on ADCC epitopes, suggesting significant immune pressure applied from ADCC responses [37], which has also been supported in animal models [38, 39]. Diverse vaccine regimens have likewise supported a role of antibodies with potent effector function in accomplishing protection from infection [15]. In the context of low HIV-specific IgA, ADCC activity was associated with reduced risk of infection among RV144 HIV-1 vaccine recipients [40]. Additional analyses of this trial have observed that polyfunctional antibodies were induced [41]. Antibodies to the V1V2 variable loops [42] that elicited complement deposition [43], and IgG3 antibodies [44], which show elevated phagocytic activity [45], were also correlated with reduced risk of infection. Consistent with recent NHP studies [21, 22], the phagocytic activity of HIV-specific antibodies, along with their binding to FcγRIIa, was observed to correlate with reduced risk of infection in a phase 2b trial (HVTN505) of a DNA + rAd5 vaccine expressing Envelope glycoproteins from clades A, B and C [46]. Excitingly, as in the pox-prime, protein boost RV144 vaccine, HIV-specific IgG3 responses were also associated with reduced risk of infection in this vaccine regimen [46].

While this may be rich evidence base on its face, it has been built on numerous distinct in vitro effector function assays developed to evaluate ADCC, ADCP, ADCVI and CDC activities. These assays differ in terms of the viral targets—ranging from recombinant antigen, to cells expressing envelope, to native virus; they differ in the effector cell populations employed—varying from purified to mixed populations of primary cells to tumor-derived cell lines; and they diverge in terms of activities read out—consisting of surrogate measures of transcriptional changes, other effector cell phenotypes, or direct cytolytic or virus inhibition activity [47]. Insights into which assays best reflect processes relevant to outcomes in vivo principally stand on correlative evidence, and the antibody features that underlie potent activity in each assay are poorly resolved. To address this last limitation, we have combined here a high throughput, multiplexed assessment of features of potential importance to these functions with machine learning in order to define aspects of the polyclonal antibody response associated with each of six effector activities previously determined in a systematic evaluation of four ADCC, one ADCVI, and two phagocytosis assays performed on a common set of serum samples from infected and vaccinated individuals [48].

## Methods

### Antibody samples

As previously described [48], serum antibodies were purified from a total of 130 subjects, including treated chronically HIV-1 infected subjects (n = 31), untreated chronically HIV-1 infected subjects (n = 28), elite controllers (n = 31), who are individuals that are able to suppress HIV-1 replication in the absence of antiretroviral therapy [49], and recipients of HIV-1 vaccine AIDSVAX B/B gp120 (n = 20) or placebo (n = 10) [50]. Study subjects’ clinical data are summarized in Additional file 1: Table S1. Polyclonal serum IgG antibody was separated from other serum proteins using the Melon gel IgG purification kit (ThermoFisher Scientific), as previously described [36]. Studies were approved by appropriate local Institutional Review Boards and each subject gave written informed consent.

### Effector functional assays

Six IgG effector functional assays were performed in three different laboratories. A common stock of research reagents, antigens, and cryopreserved PBMCs were provided to each laboratory unless otherwise specified. Assay signal to noise was defined as the ratio of the average assay signal observed for serum in HIV-1 infected subjects to that observed for placebo recipients.

### GTL ADCC

The GranToxiLux (GTL) ADCC assay was performed as previously reported [51]. CEM.NKR_CCR5_ target cells were coated with the recombinant BaL HIV-1 gp120 antigen, followed by the labeling with GranToxiLux (GTL; Oncolmmunin, Inc), which fluoresces upon cleavage by Granzyme B (GrB) released by effector cells present in heterologous PBMC from a healthy donor. ADCC-mediating antibodies in IgG from serum samples were quantified as the percent of antigen-coated cells that took up GrB. Peak activity and the area under the titration curve (AUC) was reported as the assay readouts.

### LUC ADCC

LUC ADCC assay was performed essentially as previous publication discussed [52, 53]. A vector expressing both the BaL envelope (HIV BaL.LucR.T2A.ecto/293T/17; accession number DQ318211) and the Renilla luciferase reporter gene was used to transfect the CEM.NKR_CCR5_ cell line. PBMCs were used as effector cells as previously described [54]. IgG was incubated with transfected cells and PBMCs for 6 h. ADCC-mediating antibodies induced target cell death and resulted in the reduction of virus-derived luciferase signal, which was then reported as the assay readout. Peak activity and the area under the titration curve (AUC) were reported.

### RFADCC

The rapid fluorescent ADCC (RFADCC) was performed as previously described [16]. Briefly, the CEM-NKR_CCR5_ T cell line was labeled with intracellular Carboxyfluorescein succinimidyl ester (CFSE), orange fluorescent dye PKH26, and recombinant SF162 gp120 protein. NK cells enriched from healthy donor blood with RosetteSep (Stem Cell Technologies) were used as effector cells. IgG was incubated with target cells and NK cells for 4 h at 37’C. The proportion of PKH26 + /CFSE- cells was determined by the flow cytometry and reported.

### BVADCC

The bound virion ADCC (BVADCC) was performed as previously reported [55, 56]. In brief, the CEM-NKR-CCR5 + T cell line was labeled with intracellular Carboxyfluorescein succinimidyl ester (CFSE), membrane orange fluorescent dye PKH26, and spinoculated with entry competent but replication-defective AT-2 BaL virions (Dr. Jeff Lifson, FCRC, NIH) [57–59] at 12 °C followed by incubation with serum IgG for 3 h at 37 °C. Dual dye loss of the target cells was recorded by the flow cytometry [16]. Peak activity and the area under the titration curve (AUC) were reported.

### ADCVI

The antibody-dependent cell-mediated virus inhibition (ADCVI) assay was performed as previously reported [27]. ADCVI measures the change of p24 production in the presence of antibody and FcyR-bearing effector cells. Briefly, antibody was incubated with HIV-infected CEM-NKR_CCR5_ cells and fresh PBMCs for 7 days at 37 °C. The average percent decrease of p24 produced by infected cells was measured by p24 ELISA and defined as ADCVI Activity.

### Virion phagocytosis

Phagocytosis was performed as previously described [60]. In brief, serum IgG was incubated with FITC-labeled HIV-1 US657 (GenBank U04908), followed by the addition of THP-1 effector cells. FITC-labeled virus internalized by effector cells were identified by flow cytometry. The percentage of FITC + cells was multiplied by the corresponding fluorescent intensity and reported as the assay readout. The assay background, determined by calculating the FITC + cells in the absence of Env-specific antibody, was subtracted from the signals. The average readout of replicates was reported as an assay summary measure. Although phagocytosis assays using FITC-labeled virus may be useful for assessing Fc-FcγR interactions, FITC has been shown to aggregate virions independently of antibody, raising some questions as to physiological relevance of this assay [61].

### IgG titering antigen array assay

As previously described [62], a panel of HIV-1 antigens (Additional file 1: Table S2) was printed onto glass slides in triplicate, followed by incubation with blocking reagents. IgG was diluted in a ratio between 1:100 and 1:500 and then incubated with the array. Arrays were blocked again and washed thoroughly before labeling with fluorescent Goat Anti-human IgG detection antibody. The arrays were then detected by a GenePix 4200Al (Molecular Devices) and analyzed by GenePix Pro 6.0 (Molecular Devices). The median fluorescence intensity (MFI) of each spot was recorded, and the average of the triplicates was reported as assay summary measure.

### Fc array

A customized multiplexed microsphere assay to define the characteristics of both Fv and Fc domains of purified serum antibody samples was conducted as previously described method [63]. Briefly, recombinant protein antigens (Additional file 1: Table S2) were covalently coupled to fluorescently-coded magnetic microspheres, which were incubated with dilute antibody to permit antigen binding, followed by washing and characterization of Fc domains using fluorescent detection reagents that included Fc receptor (FcR) tetramers, lectins, and secondary reagents (Additional file 1: Table S2) to identify total antibody isotypes and IgG subclasses. Data was acquired on a Bio-plex array reader (FlexMap 3D, Bio-Plex Manager 5.0, Bio-Rad). The net median fluorescence intensity (MFI) was reported.

### Data visualizations

HIV-specific antibody features defined by the Fc Array were centered and scaled. Heatmap analysis of relationships between subjects and among antibody features defined by the Fc Array and antibody effector functions was performed using the heatmap.2 function [64] in R 3.3.1 [65]. Similarities and differences in overall HIV-specific antibody response profiles defined by the Fc Array were defined by Uniform Manifold Approximation and Projection (UMAP) analysis using with the “UMAP” package [66, 67] in R.4.0.4 with default “naïve” method settings. For each subject, the two embedding coordinates were plotted using Graphpad Prism.

### Modeling antibody functions

Effector function data collected from HIV-1 infected subjects (elite controllers, and chronically infected subjects on or off therapy) were predicted by biophysical data using HIV specific antigens (gp140, gp120, gp41, non-envelope antigens and assessment of total serum IgG, IgA, and IgM) using a regularized random forest (RRF) tree algorithm [68]. Decision trees (2000) were trained to minimize regularized mean squared error and evaluated in the setting of fivefold cross-validation with the coefficient of regularization set at 0.8. Prediction accuracy was computed as the Pearson correlation coefficient between mean predicted values and experimentally observed values. Contributions of individual biophysical antibody features to prediction of functional activities were evaluated by defining the percent increase in mean square error (% Increase MSE) when individual features were permuted. Features with larger values represent those with greater importance/lower redundancy.

Model robustness was evaluated by comparison of performance of models trained with the experimental effector function data to those trained on permuted data [69], or with influenza-specific antibody response data. Influenza-specific antibody features were selected for use in this control as like HIV, influenza virus demonstrates a high level of antigenic diversity and all subjects were expected to be seropositive. In permutation tests, the model’s output values (observed effector function) were randomized and RRF models were learned under the same settings using the same input features but permuted outputs, and this process was repeated for 250 iterations. The average prediction accuracy for both permuted outputs, and for influenza-based inputs was compared to the original predictions using an unpaired two-sided student t test corrected for multiple hypothesis testing by controlling the false discovery rate to 1% using the two-stage step-up procedure of Benjamini, Krieger, and Yekutieli [70].

## Results

**Fig. 1:**
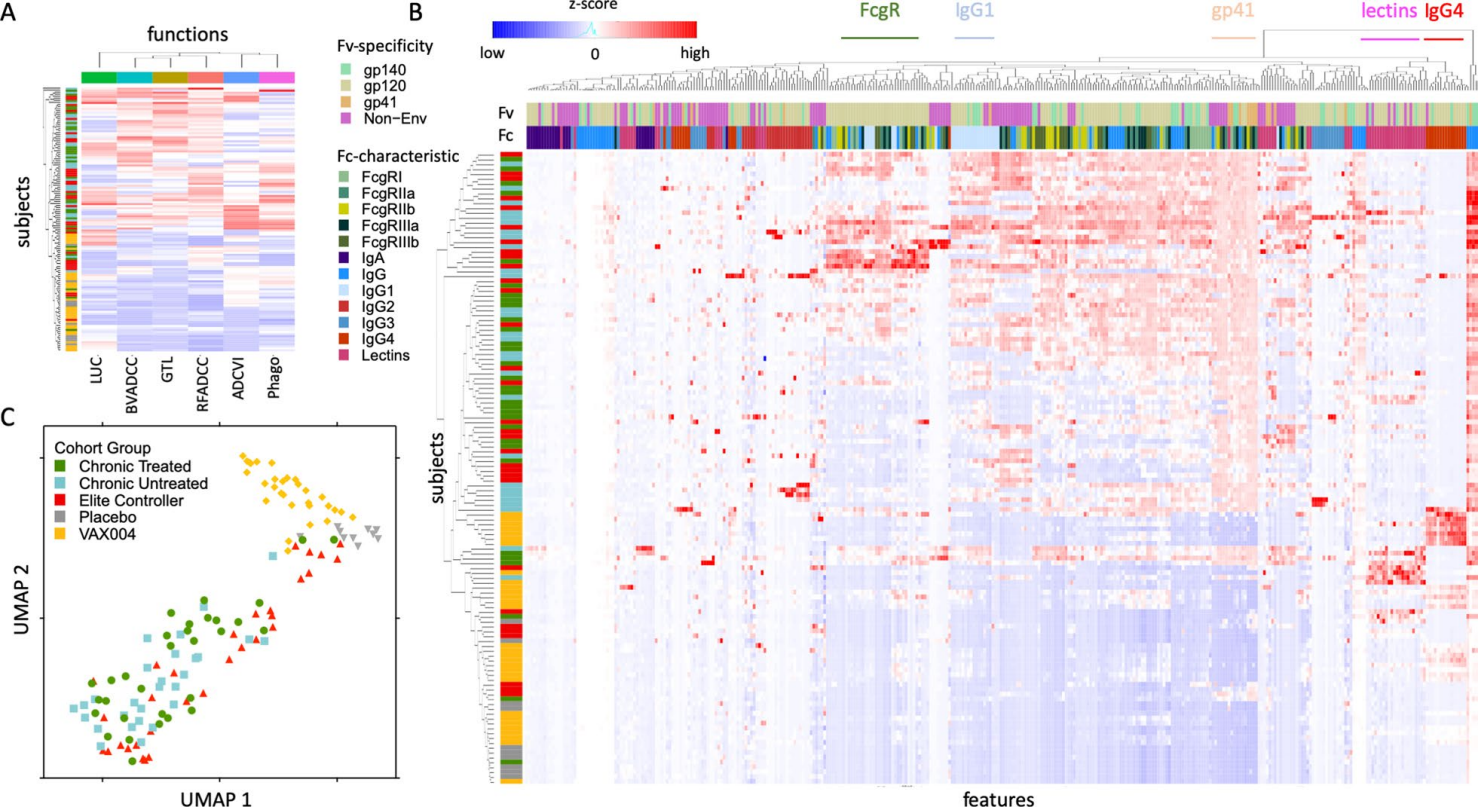
Functional and phenotypic profiles of serum antibodies. **A** Heatmap of antibody effector functions. Each column represents a specific measured effector function, and each row represents an individual subject, with subject groups indicated by the color bar at left. **B** Heatmap of HIV-specific antibody features. Each column represents a specific measured antibody feature, and each row represents an individual subject. Subject group is indicated by the vertical color bar and antibody Fv and Fc features by the two horizontal color bars. Several clusters of measures with conserved Fv specificity or Fc characteristics are labeled. Centered and scaled response magnitudes are plotted with high responses in red, low responses in blue, and median responses in white. Dendrograms indicate the hierarchical clustering of subjects and response measurements. **C** UMAP biplots representing the response profiles for each subject, colored by group. UMAP1 and UMAP2 are two calculated values representing variability of the input data

Purified polyclonal antibodies collected from chronically infected subjects on (treated) and off (untreated) anti-retroviral therapy, elite controllers, and VAX004 HIV-1 recombinant gp120 vaccine and placebo recipients were previously tested in six different IgG effector function assays conducted by different labs [48]. These assays include four antibody-dependent cellular cytotoxicity assays (GTL, LUC, RFADCC, and BVADCC), one antibody-dependent phagocytosis (Phagocytosis) assay and one antibody-dependent cell-mediated virus inhibition (ADCVI) assay. As previously reported, serum IgG from HIV-infected subjects generally showed a more robust capacity to induce diverse effector functions than that from vaccine recipients, who in turn showed elevated responses as compared to placebo recipients (Fig. 1A). A subset of HIV-infected subjects showed elevated activity across multiple ADCC assays (RFADCC, BVADCC, and GTL). Different subsets of infected subjects showed high activity in ADCVI and Phagocytosis assays. As previously reported [71], serum IgG from a subset of the elite controllers exhibited a polyfunctional profile.

The same set of purified IgG samples were also tested in two different biophysical binding assays: a microarray approach used to detect IgG responses to a set of printed antigens, and a multiplexed bead array used to define antibody isotypes, subclasses, and FcγR- and lectin-ligation profiles across diverse antigen types and viral strains. To gain a sense of the specific features that differed between subjects and groups, HIV-specific response data from the bead array was visualized in clustered heatmap form (Fig. 1B**)**. Whereas responses among placebo recipients were uniformly low across measurements, HIV-specific antibody responses among vaccine recipients and infected subjects varied considerably. Robust induction of IgG4 rarely resulted from natural infection, but was widely observed among VAX004 vaccine recipients. A subset of subjects exhibited antibodies with elevated ability to bind to lectins. This characteristic was not well correlated with other measures of the antibody response, suggesting it represents a unique distinction, the precise origin and functional consequences of which are unknown. Whereas gp41-specific responses were relatively similar in magnitude across infected subjects, responses to gp120 and gp140 showed a considerably greater degree of variability. Robust IgG2 responses were rare, and the overall magnitude of IgG antibodies was similar to IgG1 levels and antibody FcγR-binding activity. Antibodies to internal proteins, including p24, nef, integrase, and others, were also observed among infected subjects.

To better represent these complex profiles graphically, dimensionality reduction by unsupervised UMAP analysis was performed across features specific to domains of HIV-1 envelope relevant to both vaccine and infection cohorts (gp120 and gp140 only) (Fig. 1C). Whereas placebo recipients were relatively tightly clustered, other groups showed greater spread in the distribution of subjects. Antibody profiles from VAX004 recipients were clearly distinct from those observed among infected subjects. Whether treated or untreated with antiviral therapy, chronically infected subjects fell in overlapping regions of the biplot, with elite controllers appearing somewhat shifted toward the lower right edge of this distribution.

**Fig. 2:**
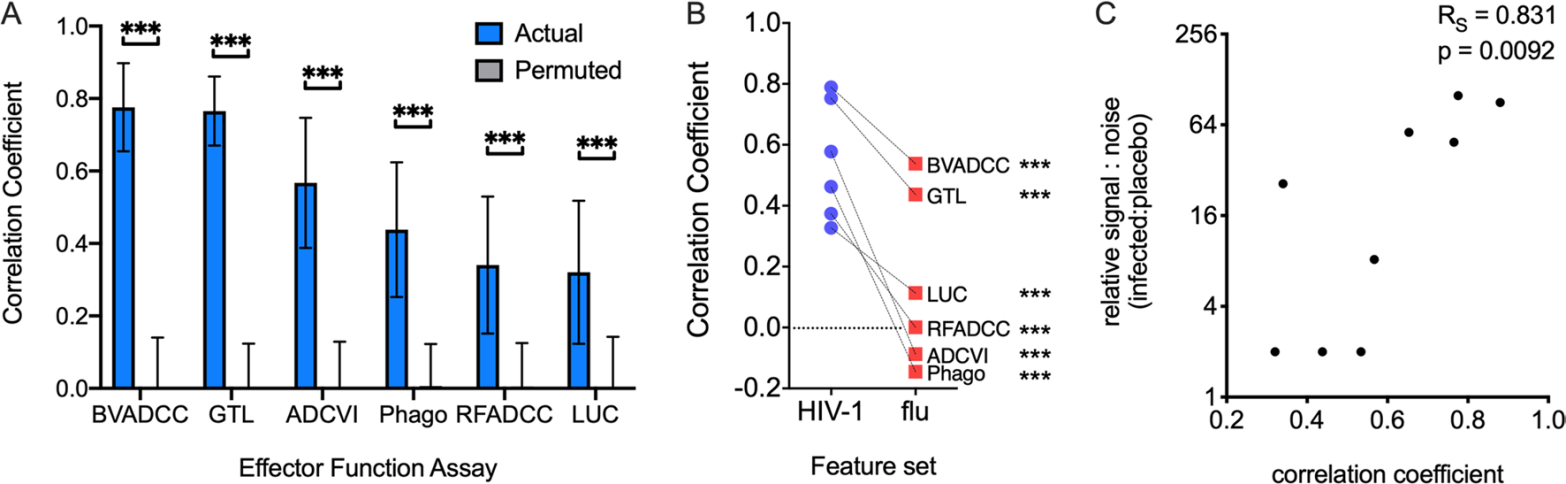
IgG effector functions are robustly predicted by HIV-specific antibody biophysical features. **A** Correlation coefficients between observed values and predicted values resulting from models learned from actual (blue) and permuted (gray) data when HIV-specific antibody biophysical features were used to predict effector functions via a regularized random forest tree model in the setting of repeated cross-validation. Bars and whiskers denote the mean and standard deviation. **B** Accuracy of models learned from HIV-specific antibody features (blue) as compared to influenza-specific antibody features (red). Statistical significance was defined by t-test adjusted for multiple hypothesis testing (***p < 0.001). **C**. Scatterplot of prediction quality (correlation coefficient) and assay signal to noise ratio. Spearman correlation coefficient and statistical significance reported in inset

The varying antibody responses observed among groups and between individuals provided the opportunity to attempt to define which antibody features were associated with each antibody effector function among HIV-infected subjects. To accomplish this goal, a supervised RRF machine learning model was trained to predict measured effector functions using IgG biophysical measurements. Predictive performance in the setting of fivefold cross-validation was evaluated across 20 replicates. Whereas models trained on actual data achieved robust prediction, defined as the degree of correlation between observed and predicted activity, models trained on permuted data had no predictive value (Fig. 2A). Similarly, when influenza rather than HIV-1 antibody features were used to train the model, accuracy was significantly degraded, most often to that expected at random (Fig. 2B). While all six IgG effector functions were robustly predicted, model accuracy varied considerably. This variation was strongly associated with the signal to noise profiles inherent to each assay in this data set (Fig. 2C).

**Fig. 3:**
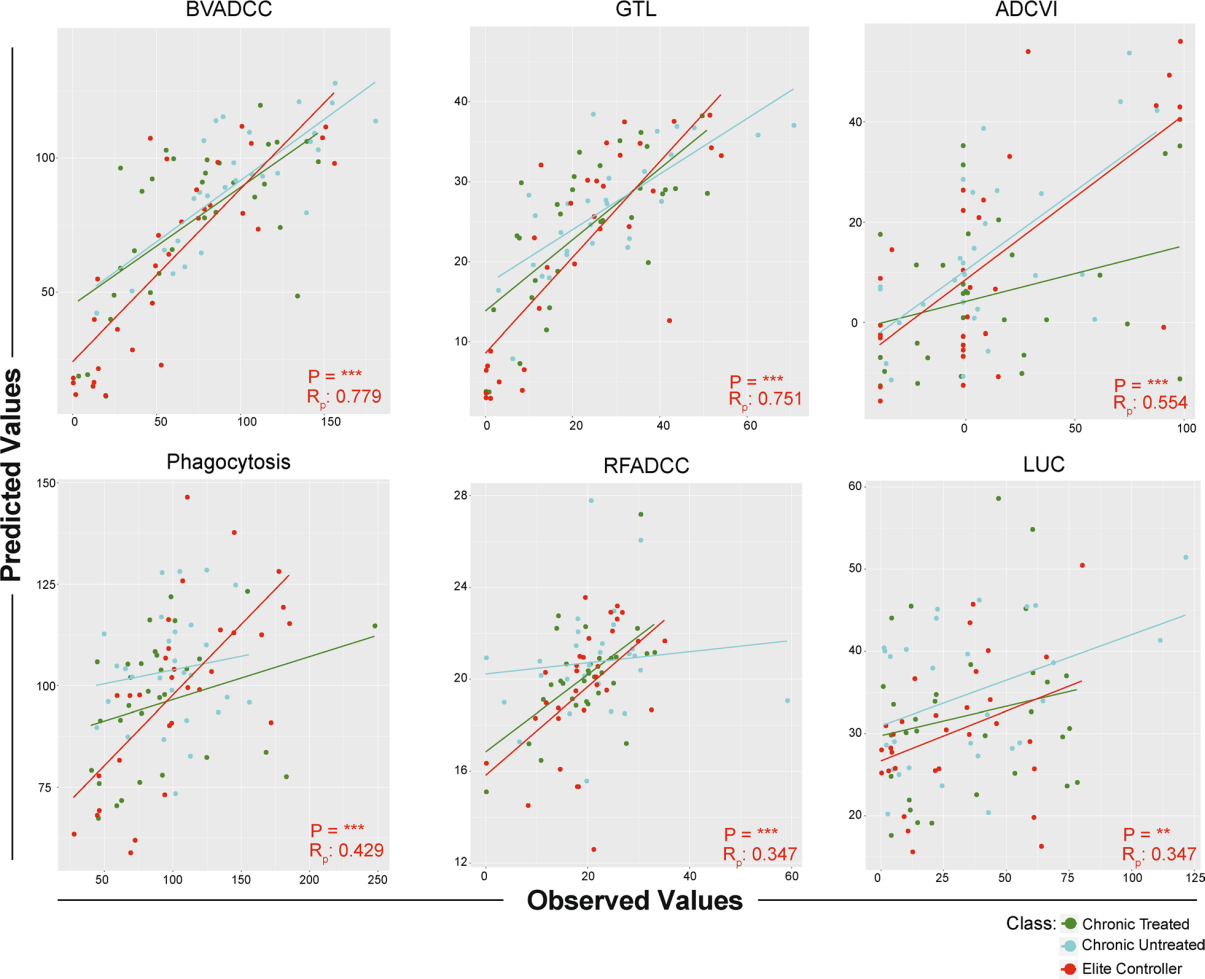
IgG effector functions predicted by IgG biophysical features in infected subjects. Scatterplots depicting the degree of correlation between of predictions of each effector function and observed activity for each HIV-infected subject. Subjects are colored by treatment and controller status. Inset reports Pearson correlation coefficients (R_P_) and statistical significance (**p < 0.01, ***p < 0.001). Colored lines indicate the best fit for each subject group

**Fig. 4:**
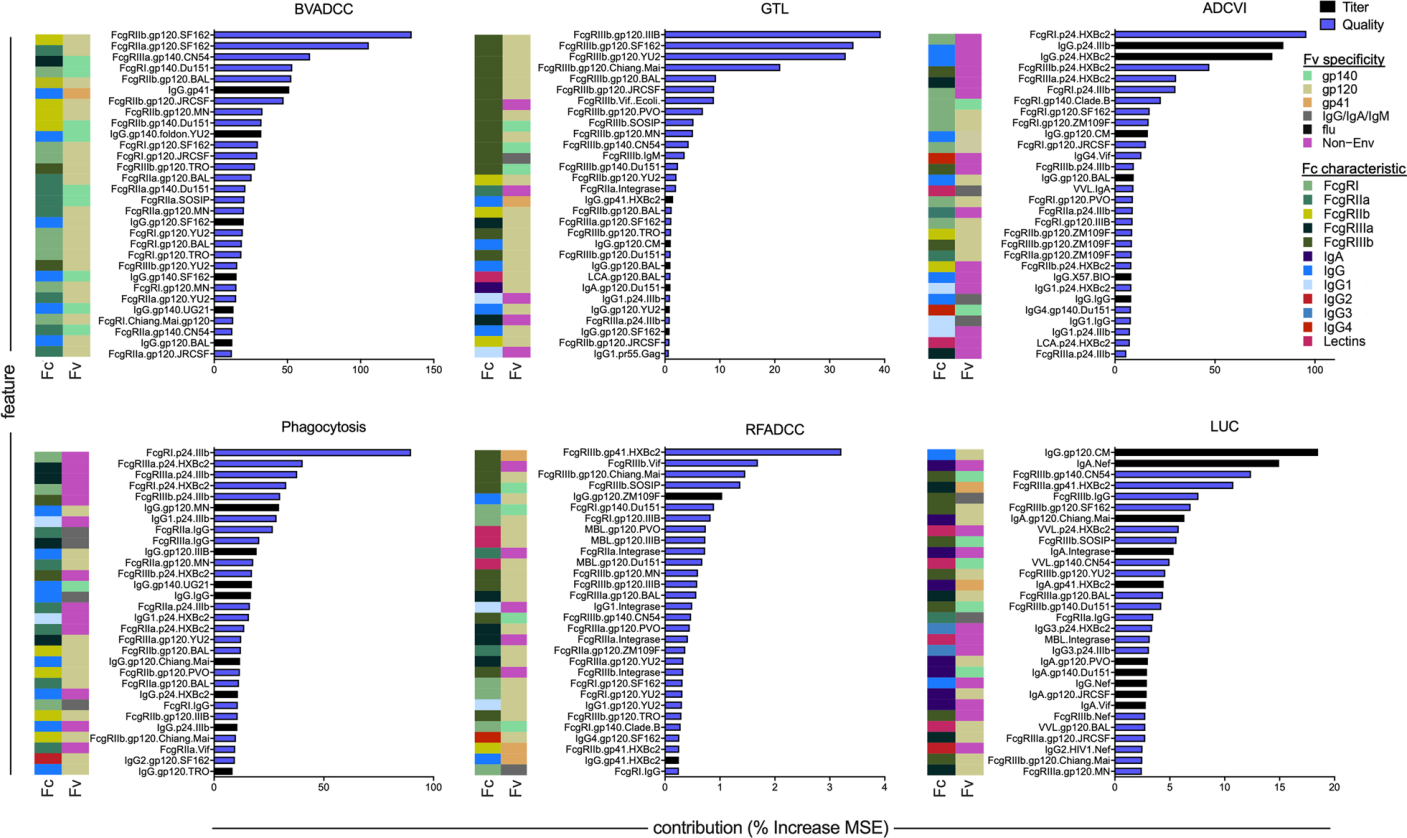
Antibody features contributing to prediction of each function. Bar chart depicts the identity and extent of contribution of the top 30 biophysical measurements in models of each effector function. Feature contributions are reported as the percent increase in model mean squared error (MSE), which quantifies the degradation in model performance when a given feature is permuted. Feature contribution bars are color coded by feature type: features that characterize the magnitude of total IgG or IgA specific for each antigen are denoted as response magnitude measures (titer, black), and those that capture IgG subclass, FcγR, and lectin binding profiles are denoted as qualities of the response (quality, blue). Color bars at left indicate the Fv specificity and Fc characteristic of each feature

The degree of agreement between predicted and actual activity in each assay for each HIV-infected subject group suggested that, in general, models predicted activity among each group equivalently well (Fig. 3). Having established robust predictive performance across assays and subject groups, we next sought to define the features supporting each model (Fig. 4). The relative importance of contributing features, whether they represented a strictly quantitative (IgG titer) or qualitative (Ig subclasses or Fc domain characteristics) aspect of the humoral response, was defined for each function. Across effector assays, distinctions in the antigen-specificity and Fc domain characteristics relevant to each activity were readily apparent.

GTL and BVADCC assays were the most accurately predicted, and both aim to measure antibody-mediated cellular cytotoxicity. While they were among the most highly correlated pairs of assays, the major supporting features turned out to be very different. For GTL assay activity, gp120-specific antibody ligation of FcγRIII features were primary supporting features, consistent with the reliance of this assay on gp120-coated target cells, and NK cells as effectors. In contrast, a broader diversity of envelope-specific antibody ligation of FcγRII contributed primarily to the prediction of BVADCC results, and only three FcγRIII features were selected among the top 30 features. In terms of antigen recognition, given the use of inactivated virions in this assay, contributions from gp41- and gp140-specific antibodies are likely to have direct mechanistic relevance. In terms of Fc domain characteristics, a strong dependence on FcγRII, more widely associated with phagocytosis and a diversity of effector cells, was noted. Interestingly, subsequent experiments have demonstrated that this assay, which employs unfractionated PBMCs, actually primarily assesses activity mediated by monocytes, and which is mechanistically dependent on FcγRII [72, 73].

Though not predicted as well as these other ADCC assays, models of RFADCC assay activity showed a strong dependence on FcγRIII, consistent with the use of purified NK cells as effectors, and its readout of cell permeabilization dependent on GrB activity driven by FcγRIII ligation. One attribute common to each of these assays is their strong reliance on the measures of antibody quality (FcR-related features), as compared to measures of response magnitude alone (anti-human IgG detection reagents), indicating that titer-related measurements were less important than Fc-specific measurements in predicting these functions.

For ADCVI and phagocytosis assays, p24-specific antibodies were major contributing features. While both assays evaluated responses in the context of replication-competent virus, meaning that p24 was present, it is not clear how a mechanistic role for p24-specific antibodies in restricting viral outgrowth (ADCVI) or driving phagocytosis (fluorescent virion uptake) might be mediated. For ADCVI, which showed a clear degradation in model accuracy when p24 features were omitted (R_P_ = 0.55 versus 0.47 without p24 features, p < 0.05), one possibility raised to by this model is that p24-specific antibodies present in each sample may interfere with the p24 quantitation that serves as the measure of virus replication read out by the assay. This possibility does not exist for the phagocytosis assay, which does not involve p24 quantitation, and for which omission of p24 features still resulted in a small but statistically significantly reduction in accuracy (R_P_ = 0.43 versus 0.39 without p24 features, p = 0.10). Interestingly, and unlike other functions, model performance for phagocytosis was superior among elite controllers (elite controllers R_P_ = 0.66, p = 0.00006; chronic treated R_P_ = 0.06, p = 0.05; chronic untreated R_P_ = 0.16, p = 0.4), who are known to exhibit higher levels of p24-specific antibodies [74, 75] as compared to chronically infected subject groups. Overall, while direct mechanistic contributions cannot be determined without further experiment, the data driven approach employed linked the otherwise unrelated observations of better ADCVI activity observed in elite controllers [36] and long term non-progressors [25] and their elevated p24-specific antibody levels [74, 75]. Coupled to observations related p24-specific responses to protection made in the setting of protective vaccines [76], these results suggest a potential avenue for further mechanistic evaluation [77].

Lastly, led by a titer measurement (IgG specific to gp120 CM), a number of envelope-specific FcγRIII features contribute to predictions in the LUC assay. Intriguingly, despite the use of IgA depleted samples, a number of IgA features, including responses specific to internal proteins such as Nef and Integrase also made contributions. While mechanistic contributions cannot be excluded, interpretation of model features must also consider assay reproducibility and model performance quality. The best performing models provided new, mechanistically relevant insights, and the less well-performing models can be considered to point to both the limitations of modeling noisy biological data, as well as to the complexity of antibody effector functions and the challenges to developing robust in vitro assays for their assessment.

## Discussion

Titers of antigen-specific binding antibodies and neutralizing antibodies are often useful but not entirely sufficient measures to fully explain therapeutic benefit or vaccine efficacy across a wide variety of pathogens [76, 78–84]. Given the challenges in achieving high titers of broadly neutralizing antibodies, the discrepancy between antibody quantity and antibody quality have posed special challenges to development of an effective HIV-vaccine. Because both NHP and human immune correlate studies of experimental vaccines have suggested the importance of antibody effector functions [46, 85–87], these functions offer an additional means to restrict infection, thereby motivating interest in defining how different aspects of antibody quality and quantity contribute to diverse activities. Potent effector functions are associated with a set of unique antibody characteristics, including N-linked glycosylation of the Fc domain, antibody titer, immune complex size/geometry, epitope specificity, binding avidity, and IgG subclasses [56, 88–92].

While these in vitro antibody activities have shown associations with protection from HIV-1 infection, slower progression of AIDS, or lower viral loads in both humans and nonhuman primates, most of the current effector function assays utilize pre-defined antigens and cell lines, which do not fully recapitulate the complexity of the in vivo immune responses. The variation of individual effector cell populations and genetic polymorphisms in Fc receptors all pose additional challenges to the interpretation of results in simplified in vitro antibody effector function assays. These and other host-specific factors add a layer of further functional diversity. Despite the inability of correlative relationships to provide direct evidence of mechanism of action, it may be important to apply multiple and orthogonal assays to acquire a comprehensive understanding of the spectrum of possible *in-vivo* antiviral antibody activities.

Here, to dissect this rich landscape, supervised machine learning models were trained to predict effector function among HIV-infected subjects using antibody profiling data. The two most accurately predicted effector function assays, GTL, and BVADCC, were highly correlated with each other, but predicted by a distinct set of response features. In general, these and other functional assays chiefly relied upon measures of antibody quality (FcγR binding propensity and IgG subclass) rather than antibody quantity (antigen-specific IgG titer), and exposed the role of an unanticipated receptor in one of the assays that was validated experimentally. Collectively, consistent with prior studies [75, 76, 91, 93], these results emphasize the value of specific quality attributes of humoral responses in inducing potent effective effector functions.

While there was considerable diversity in the antibody profiles among infected subjects, responses in vaccinees were even more distinct. In particular, IgG4 responses were strikingly elevated among VAX004 vaccine recipients as compared to responses observed among infected individuals, as was previously noted for VAX003 [44]. Such profound differences point to the plasticity of the immune response. Better understanding of how class switch recombination is regulated has clear value to both vaccine design and development of novel approaches to antibody-driven allergic and autoimmune responses.

## Conclusions

Given the growing number of studies in the past decade that have revealed the essential role of Fc and FcγR-mediated effector activities in both animal and human studies [12], coupled to the diversity of assays used to characterize these activities [47], we sought to marry previously published results for a set of cell-based FcγR-mediated effector function assays [48] to biophysical antibody profiling assays on a common set of serum samples from HIV infected and vaccinated individuals. Intrigued by acquiring a deeper resolution of specific antibody features associated with complementary effector functions, this study discovered more differences than similarities among the different assays. By using machine learning methods to relate activity observed in each assay to underlying characteristics of the humoral response as defined by two high-throughput, multiplexed profiling assays, we found that assays with good signal to noise could be robustly predicted from HIV- but not permuted or influenza-specific antibody profile data. Further, even among the most highly correlated assays, predictions were driven by unique combinations of response characteristics that could be mechanistically confirmed. While these results do not enable identification of the activities most relevant to antibody-mediated protection from HIV infection in vivo, they offer further insights into the diversity of effector cell types and associated antibody phenotypes that may contribute to viral blockade and restriction.

## Supplementary Information

**Additional file 1: Table S1.** Clinical characteristics of HIV+ Subject Groups. Mean and interquartile ranges (IQR) of ages, sex, viral load, and CD4 T cell counts for untreated, treated, and elite controllers. Presence or absence of protective HLA-B alleles were balanced across groups. **Table S2.** Antigen specificities and Fc detection reagents.

## Data Availability

The datasets analyzed in this study are available from the corresponding author on reasonable request.
