# Supplementary material for: Diverse antiviral IgG effector activities are predicted by unique biophysical antibody features
Source: Retrovirology. 2021 Oct 30;18:35. doi: 10.1186/s12977-021-00579-9 (PMC8557579; doi:10.1186/s12977-021-00579-9)
Supplement: Supplementary file 1 — Additional file 1: Table S1. Clinical characteristics of HIV+ Subject Groups. Mean and interquartile ranges (IQR) of ages, sex, viral load, and CD4 T cell counts for untreated, treated, and elite controllers. Presence or absence of protective HLA-B alleles were balanced across groups. Table S2. Antigen specificities and Fc detection reagents. [file 12977_2021_579_MOESM1_ESM.docx]

**Table S1.** Clinical characteristics of HIV+ Subject Groups. Mean and interquartile ranges (IQR) of ages, sex, viral load, and CD4 T cell counts for untreated, treated, and elite controllers. Presence or absence of protective HLA-B alleles were balanced across groups.

|  | **Age (years)** | **Sex** | **Viral load** | **CD4 T cell count** |
| --- | --- | --- | --- | --- |
|  | Mean (IQR) | Female (%) | Mean (IQR) | Mean (IQR) |
| **Untreated** | 35 (30-39) | 32% | 34,000 (3,728-55,000) | 480 (300-680) |
| **Treated** | 35 (31-39) | 52% | 66 (48-75) | 460 (170-620) |
| **Controller** | 34 (31-38) | 50% | 85 (48-75) | 824 (700-920) |

**Table S2.** Antigen specificities and Fc detection reagents.

| **Bead Array** | | | | **Printed Slide Array** | | | |
| --- | --- | --- | --- | --- | --- | --- | --- |
| **Detection Reagents** | | **Fv specificity** | | **Detection Reagent** | | **Fv specificity** | |
| isotype | IgA | HIV | gp120.93TH975 | isotype | IgG | HIV | gp120.YU2 |
|  | IgG |  | gp120.BAL |  |  |  | gp120.D368R.YU2 |
| subclass | IgG1 |  | gp120.CM235 |  |  |  | gp140.foldon.YU2 |
|  | IgG2 |  | gp120.Du151 |  |  |  | gp41 |
|  | IgG3 |  | gp120.Du156.12 |  |  |  | gp120.JRCSF |
|  | IgG4 |  | gp120.IIIB..CHO. |  |  |  | gp120.D368R.JRCSF |
| Fcg Receptors | FcgRI |  | gp120.JRCSF |  |  |  | gp120.CM |
|  | FcgRIIa |  | gp120.MN |  |  |  | gp120.JRFL |
|  | FcgRIIb |  | gp120.PVO |  |  |  | g120.N332A.JRFL |
|  | FcgRIIIa |  | gp120.SF162 |  |  |  | gp120.BaL |
|  | FcgRIIIb |  | gp120.TRO |  |  |  | gp120.BaL |
| Lectins | LCA |  | gp120.YU2 |  |  |  | gp120.93TH975 |
|  | MBL |  | gp120.ZM109F |  |  |  | gp120.CN54 |
|  | PNA |  | gp120.Chiang.Mai |  |  |  | gp120.IIIB |
|  | SNA |  | gp140.Clade.B |  |  |  | gp120.96ZM651 |
|  | VVL |  | gp140.CN54 |  |  |  | gp140.UG37 |
|  |  |  | gp140.Du151 |  |  |  | gp140.CN54 |
|  |  |  | SOSIP |  |  |  | gp140.UG21 |
|  |  |  | gp41.HXBc2 |  |  |  | gp140.SF162 |
|  |  |  | p24.HXBc2 |  |  |  | 179.4.BIO |
|  |  |  | p24.IIIb |  |  |  | 57.BIO |
|  |  |  | pr55.Gag |  |  |  | RSC3 |
|  |  |  | Integrase |  |  |  | deltRSC3 |
|  |  |  | Nef |  |  |  |  |
|  |  |  | Rev |  |  |  |  |
|  |  |  | Vif |  |  |  |  |
|  |  | other virus | Flu.BNA |  |  |  |  |
|  |  |  | Flu.MNA |  |  |  |  |
|  |  |  | Flu.N2 |  |  |  |  |
|  |  |  | HA.Brisbane.10.07 |  |  |  |  |
|  |  |  | HA.NewCal.20.99 |  |  |  |  |
|  |  |  | HA.Wisconsin.67.05 |  |  |  |  |
|  |  |  | HAV.P2C.P3a |  |  |  |  |
|  |  |  | HCV.E2 |  |  |  |  |
|  |  |  | HSV1.gG |  |  |  |  |
|  |  | total Ig Isotype | IgA |  |  |  |  |
|  |  |  | IgG |  |  |  |  |
|  |  |  | IgM |  |  |  |  |
